# Distinct Signaling Pathways Between Human Macrophages and Primary Gingival Epithelial Cells by *Aggregatibacter actinomycetemcomitans*

**DOI:** 10.3390/pathogens9040248

**Published:** 2020-03-27

**Authors:** Ellen S. Ando-Suguimoto, Manjunatha R. Benakanakere, Marcia P.A. Mayer, Denis F. Kinane

**Affiliations:** 1 Department of Microbiology, Institute of Biomedical Sciences, University of São Paulo, São Paulo 05508-020, Brazil; mpamayer@icb.usp.br; 2 Department of Periodontics, School of Dental Medicine, University of Pennsylvania, Philadelphia, PA 19104, USA; 3 Department of Periodontology, School of Dental Medicine, University of Geneva Faculty of Medicine, 12114 Geneva, Switzerland; dfkinane@outlook.com

**Keywords:** *A. actinomycetemcomitans*, inflammasome, immune response, periodontal disease

## Abstract

In aggressive periodontitis, the dysbiotic microbial community in the subgingival crevice, which is abundant in Aggregatibacter actinomycetemcomitans, interacts with extra- and intracellular receptors of host cells, leading to exacerbated inflammation and subsequent tissue destruction. Our goal was to understand the innate immune interactions of A. actinomycetemcomitans with macrophages and human gingival epithelial cells (HGECs) on the signaling cascade involved in inflammasome and inflammatory responses. U937 macrophages and HGECs were co-cultured with A. actinomycetemcomitans strain Y4 and key signaling pathways were analyzed using real-time PCR, Western blotting and cytokine production by ELISA. A. actinomycetemcomitans infection upregulated the transcription of TLR2, TLR4, NOD2 and NLRP3 in U937 macrophages, but not in HGECs. Transcription of IL-1β and IL-18 was upregulated in macrophages and HGECs after 1 h interaction with A. actinomycetemcomitans, but positive regulation persisted only in macrophages, resulting in the presence of IL-1β in macrophage supernatant. Immunoblot data revealed that A. actinomycetemcomitans induced the phosphorylation of AKT and ERK1/2, possibly leading to activation of the NF-κB pathway in macrophages. On the other hand, HGEC signaling induced by A. actinomycetemcomitans was distinct, since AKT and 4EBP1 were phosphorylated after stimulation with A. actinomycetemcomitans, whereas ERK1/2 was not. Furthermore, A. actinomycetemcomitans was able to induce the cleavage of caspase-1 in U937 macrophages in an NRLP3-dependent pathway. Differences in host cell responses, such as those seen between HGECs and macrophages, suggested that survival of A. actinomycetemcomitans in periodontal tissues may be favored by its ability to differentially activate host cells.

## 1. Introduction

Periodontitis is an infectious inflammatory disease that leads to the destruction of tooth-supporting tissues by an imbalanced immune response [1,2,3,4,5]. The inflammatory process is induced by a dysbiotic microbial community, with the Gram-negative facultative species *Aggregatibacter actinomycetemcomitans* associated the rapid progression rate of periodontitis, which was previously denominated localized aggressive periodontitis and is now classified as molar/incisor pattern periodontitis [6,7]. *A. actinomycetemcomitans* is also associated with endocarditis [8] and may play a role in cardiovascular disease and arthritis [9,10]. The dysbiosis induced by *A. actinomycetemcomitans* may be the result of an immunological palsy induced by its virulence factors, such as leukotoxin (Ltx) and cytolethal distending toxin (Cdt) [11,12], which are associated with its ability to invade non-phagocytic cells [13]. 

During infection, the immune response is induced by microbial-associated molecular patterns (MAMPS), which are recognized by pattern-recognition receptors (PRRs) in eukaryotic cells. PRRs are found extracellularly as Toll-Like Receptors (TLRs) and are expressed on the cell surface, with their stimulation by MAMPS resulting from the activation of NF-κB, MAPK and IRF signaling pathways, culminating in the production of a number of cytokines, chemokines and immunomodulatory factors [14]. PRRs are also present in the cytoplasm and are here called Nod-Like Receptors (NLRs), with nucleotide binding domain/leucine rich repeats, including NLRP1, NLRP3, NLRC4, NOD1, NOD2, and AIM2 receptors; these detect intracellular microorganisms and their products [15,16,17]. Although both pathogenic and commensal microbes are recognized by PPRs, pathogens often induce the production of endogenous danger signals (danger-associated molecular patterns (DAMPs)) [18]. The cytosol senses DAMPS via NLRs, leading to the formation of multiprotein cytoplasmic complexes called inflammasomes [19]. These complexes activate caspase-1, which results in the release of mature interleukin-1β (IL-1β) and interleukin-18 (IL-18), thereby inducing pyroptosis and apoptosis [20]. 

*A. actinomycetemcomitans* is recognized by Toll-Like Receptor 4 (TLR4) and TLR2 [21]. Gingival epithelial cells (GECs) are the first defense barrier against pathogens in periodontal tissues; *A. actinomycetemcomitans* adheres to and invades epithelial cells [22]. The interaction of *A. actinomycetemcomitans* with GECs induces the expression of ICAM-1, TNF, GM-CSF, IL-6 and IL-8 and causes apoptosis in monocytes mediated by interaction with TLR2 [22,23,24]. Furthermore, macrophages infected with *A. actinomycetemcomitans* secrete IL-1β [25,26] and IL-18, a response which is associated with the purinergic receptor P2X_7_, an endogenous danger signal receptor [27].

Inflammasome activation may play a key role in periodontitis. The expression of NLRP3, which is involved in inflammasomes, is higher in chronic and aggressive periodontitis gingival tissues than in healthy tissues, especially at the periodontal epithelium layer [28]. In periodontal disease, NLRP3 salivary levels are higher in aggressive periodontitis cases compared to periodontally healthy subjects [29]. 

*A. actinomycetemcomitans* cytolethal distending toxin (AaCdt) was shown to be involved in NLRP3 activation in THP-1 monocytes and release of mature IL-1β [30], but other bacterial factors may be also associated with this response to *A. actinomycetemcomitans,* since infection of human monocytes with leukotoxin- and Cdt-deficient strains still resulted in upregulation of NLRP3, IL-1β and IL-18 expression [31]. *A. actinomycetemcomitans* was also shown to activate the inflammasome pathway in nonimmune cells. NRLP3 upregulation and secretion of mature IL-1β and IL-18 were observed in human osteoblastic MG63 cells upon exposure to *A. actinomycetemcomitans*, leading to apoptosis [32]. Furthermore, NOD1 and NOD2 were activated in human embryonic kidney cells in the presence of *A. actinomycetemcomitans* [33].

The response to a pathogen depends not only on the stimulus, but also on the cell type; however, there are no data regarding whether *A. actinomycetemcomitans* is able to activate inflammasomes in epithelial cells, as reviewed previously [34]. Given the significance of gingival epithelial cells and macrophages in aggressive periodontitis, the present study evaluated the signaling network initiated by *A. actinomycetemcomitans* in gingival epithelial cells and macrophages and, consequently, the induction of immune and inflammasome responses.

## 2. Results

*A. actinomycetemcomitans* is associated with localized aggressive periodontitis, however, the molecular mechanisms of the innate immune response in distinct myeloid and nonmyeloid cells of the oral cavity are unknown. We set out to understand the differential activation immune vs. nonimmune cells by *A. actinomycetemcomitans*. We stimulated the U937 cell line to differentiate into macrophages and primary human gingival epithelial cells (HGECs) via *A. actinomycetemcomitans* Y4 at different time points to determine the innate immune responses. The innate immune genes, in particular *TLR2, TLR4* and *NLRP3,* were upregulated in U937 macrophages co-cultured with *A. actinomycetemcomitans* after two and three hours of incubation, where *NLRP3* doubled its expression compared to the control after two hours of co-culture and was downregulated after four and eight hours, whereas *NOD2* mRNA reached maximum levels after 8 h of stimulation, increasing expression by two times. On the other hand, infection of HGECs with *A. actinomycetemcomitans* did not result in altered transcription profile of genes encoding these receptors (Table 1 and Table 2). 

**Figure 1:**
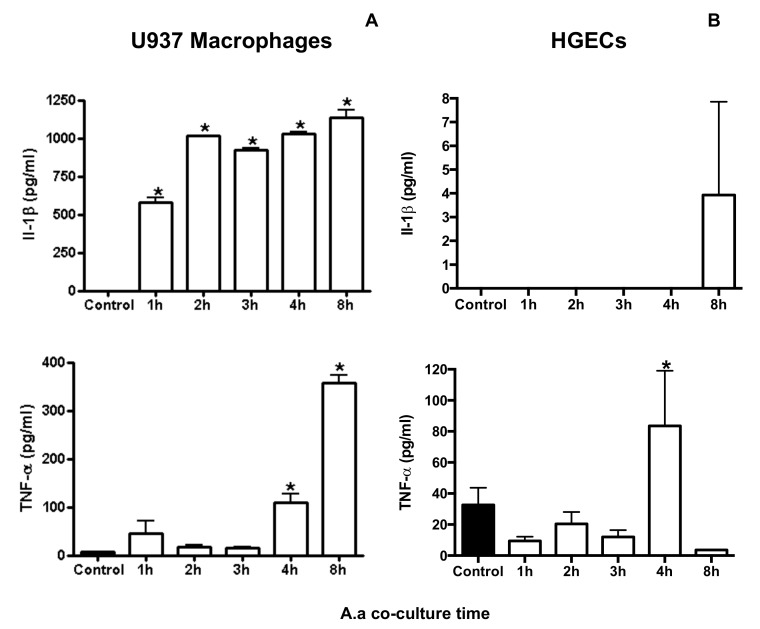
Effect of co-culture of *A. actinomycetemcomitans* strain Y4 (Multiplicity of infection (MOI) 1:100) with U937 macrophages (**A**) or HGECs (**B**) for 1, 2, 3, 4 and 8 h on the levels of IL-1β and TNF-α levels in cell supernatants. Control consisted of infected cells at 0 h. Data (pg cytokine/mL) are presented as mean ± SD representative of three independent experiments. * Statistically significant difference in comparison with control (ANOVA–Tukey’s, p < 0.05).

Cytokine gene expression, *IL-1*β, *IL-18* and *TNF* mRNA levels, increased after co-culture of macrophages with *A. actinomycetemcomitans* (Table 2). On the other hand, the interaction of *A. actinomycetemcomitans* with HGECs resulted in a small increase in *IL-1*β and *IL-18* mRNA levels after one hour of co-culture, and decreased at later time points (Table 1). *TNF* mRNA levels increased after two hours of co-culture of *A. actinomycetemcomitans,* with U937 macrophages reaching the peak after three hours of co-culture (134 times increase), and after three and four hours with HGECs (2 and 3.72 times increase, respectively). Production of IL-1β increased after *A. actinomycetemcomitans* challenge in U937 macrophages but not in HGECs. The *A. actinomycetemcomitans* challenge induced production of TNF in U937 macrophages after two hours, whereas in HGECs, TNF was induced after a prolonged period (Figure 1). 

**Figure 2:**
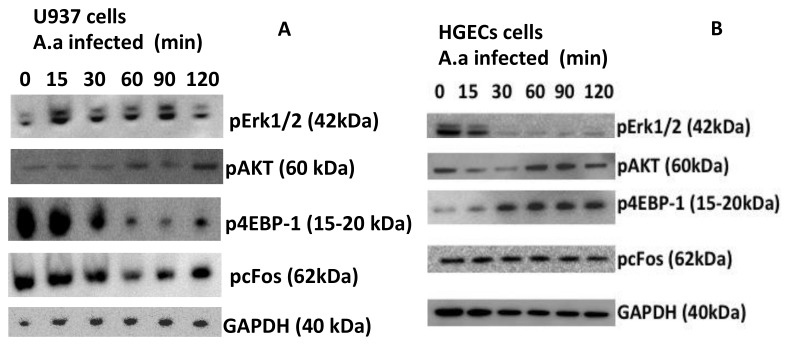
Different pathways are activated by *A. actinomycetemcomitans* in macrophages and HGECs. Western blot was used to evaluate the phosphorylation of ERK1/2, 4EBP-1, cFos and AKT in U937 macrophages (**A**) and in HGECs (**B**) after infection with *A. actinomycetemcomitans* strain Y4 (MOI 1:100) at different time points. GAPDH was used as the control. The data shown are representative of three independent experiments.

Since transcription of *TLR2, TLR4 and NLRP3* was upregulated in U937 macrophages, we determined the activation of signaling molecules downstream of TLRs. Signaling pathway analysis indicated that NF-κB was activated (phosphorylation of serine 32 in IκB-α) and pro caspase-1 was induced and cleaved in *A. actinomycetemcomitans* infected-macrophages (Figure 2A). Infection with *A. actinomycetemcomitans* induced activation of ERK1/2 (phosphorylation of T 202/Y204 residues in ERK1) after 15 min of co-culture and AKT (phosphorylation serine 473 in AKT) after 120 min of co-culture. However, p4EBP1 and pcFos levels did not increase in *A. actinomycetemcomitans*-infected macrophages. 

Interestingly, signaling induced by *A. actinomycetemcomitans* was distinct in HGECs. In these epithelial cells, AKT and 4EBP1were phosphorylated after stimulation with *A. actinomycetemcomitans,* whereas ERK1/2 was not phosphorylated (Figure 2B). On the other hand, the phosphorylation of Serine 473 (Ser 473), indicative of AKT activation, was not observed until 60 min of stimulation with *A. actinomycetemcomitans*; these data indicated that AKT activation was under the levels observed in noninfected cells at the early stages of co-culture.

**Figure 3:**
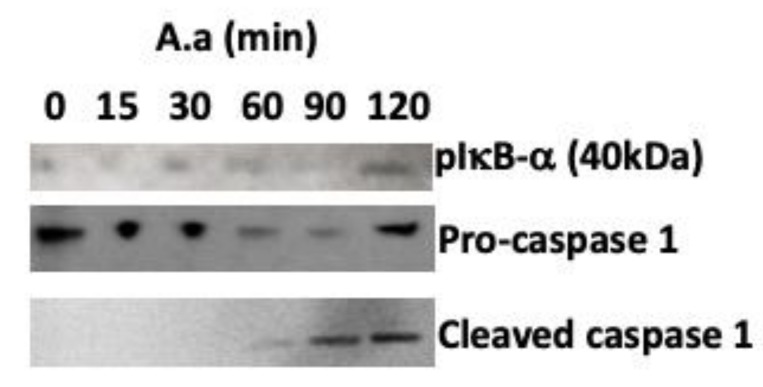
Western blot image showing increased levels of phosphorylated IκB (plkB-α), (indicative of NF-κB activation), decreased levels of procaspase-1 and increased levels of cleaved caspase-1 (indicative of inflammasome activation in U937 macrophages) after co-culture of *A. actinomycetemcomitans* strain Y4 (MOI 1:100) at different time points. GAPDH was used as the control. The data shown are representative of three independent experiments.

IL-1β and TNF-α transcription and protein levels (Table 2 and Figure 1) were increased in *A. actinomycetemcomitans*-infected macrophages within a few hours of incubation, mainly because of the activation of NF-κB in these cells (Figure 3). These results suggested that, in macrophages, NF-κB activation by *A. actinomycetemcomitans* is dependent on AKT–ERK1/2 activation, although more profound studies should still be performed. NF-κB activation induced the release of pro-IL-1β and pro-IL-18, which, in the context of the inflammasome, are processed to their mature forms by caspase-1 in macrophages, as shown by the increased production of active caspase-1 in cells co-cultured with *A. actinomycetemcomitans* (Figure 3). The data indicated that infected macrophages exhibited *NLRP3* upregulation and release of IL-1.

**Figure 4:**
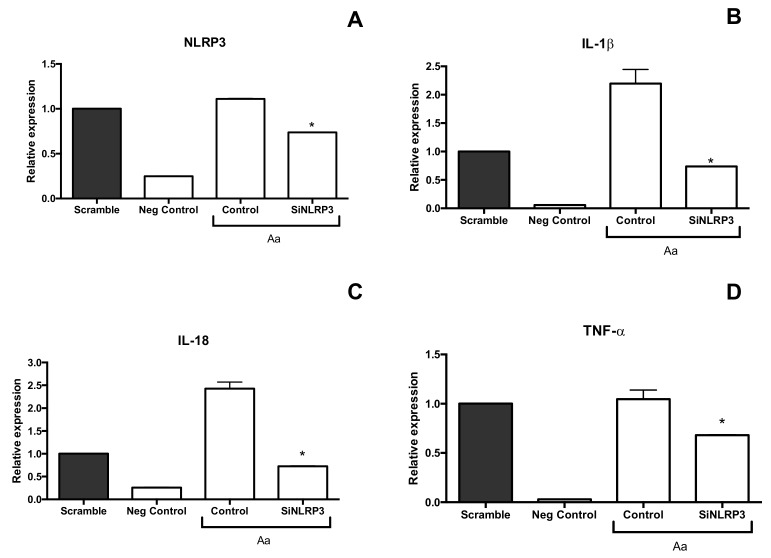
Effect of siNLRP3 silencing in *A. actinomycetemcomitans* Y4-infected U937 macrophages (MOI 1:100, 24 h of co-culture) on the relative expression of *NLRP3* (**A**), *IL-1β* (**B**)*, IL-18* (**C**) and *TNF-α* (**D**) detected by real time PCR. Scramble: Scramble control without *A. actinomycetemcomitans* (pool of nontargeting Sirna)*;* negative control: Cells without *A. actinomycetemcomitans*; control: Positive control: Cells with *A. actinomycetemcomitans* without *NLRP3* silencing; siNLRP3: Cells with silencing of *NLRP3* and co-culture with *A. actinomycetemcomitans*. * Statistically significant difference in comparison with electroporated cells with *A. actinomycetemcomitans* and scramble control (ANOVA–Tukey’s, p < 0.05). Data shown in fold-change relative to the scramble are presented as mean ± SD representative of three independent experiments.

**Figure 5:**
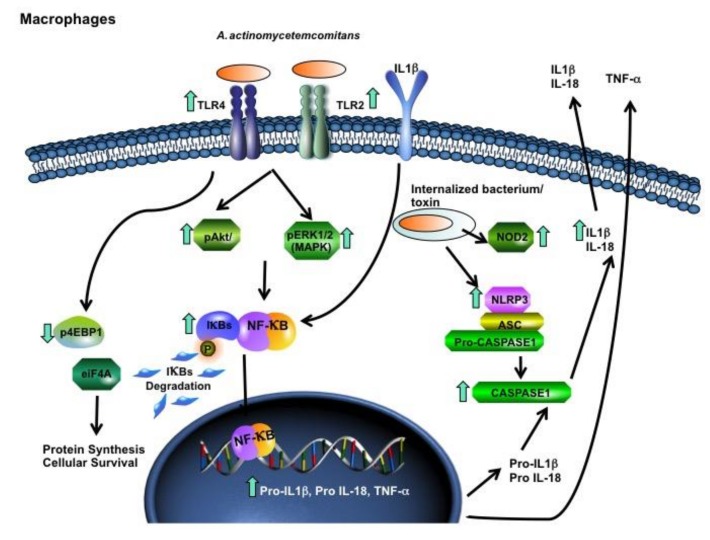
U937 macrophage response after co-culture with *A. actinomycetemcomitans* Y4. Arrows mean up- or downregulation of gene transcription for *TLR4, TLR2, NOD2, NLRP3, Pro*-*IL-1β, Pro-IL-18* and *TNF-α,* and for phosphorylation of 4EBP1, AKT, pERK1/2, IκB-α and caspase-1. *A. actinomycetemcomitans* was able to induce an inflammatory response and inflammasome activation in macrophages. Adapted from Qiagen’s website (https://www.qiagen.com/br/shop/genes-and-pathways/pathway-central/?q=).

The inflammasome response was analyzed in cells by silencing NLRP3. siNLRP3 macrophages infected with *A. actinomycetemcomitans* exhibited decreased *IL-1β* and *IL-18* mRNA levels. but not *TNF-α* (Figure 4). Taken together, *A. actinomycetemcomitans* distinctly activated innate immune and inflammasomes in myeloid cells (Figure 5) and nonmyeloid cells.

## 3. Discussion

The immune response elicited by the dysbiotic community in aggressive periodontitis induced inflammation, resulting in tissue destruction and bone resorption [35]. In this study, we analyzed two important host cell defenses, namely, a first barrier of epithelial cells and a second specialized cell, the macrophages of myeloid origin.

Human gingival epithelial cells and macrophages displayed distinct responses after challenge with *A. actinomycetemcomitans.* The expression of innate immune receptors was not altered by *A. actinomycetemcomitans* infection in HGECs, suggesting that the epithelial barrier was a weak response, resulting in a discrete increase in IL-1β and IL-18 production after one hour of co-culture and decreased expression thereafter. These data were in accordance with others demonstrating that *A. actinomycetemcomitans* did not induce IL-1β production by gingival epithelial cells [36,37]. Moreover, the increase in mRNA expression of TNF after three hours indicated that the primary HGECs respond to the bacteria stimuli, as demonstrated in immortalized OBA-09 cells [22].

On the other hand, transcription of genes encoding TLR2, TLR4, NLRP3 and NOD2 was upregulated in *A. actinomycetemcomitans-*infected macrophages, suggesting a rapid innate immune response against the pathogen. Previous studies in macrophages [38,39] and dendritic cells [40] corroborated our observation that *A. actinomycetemcomitans* leads to increased expression of TLR2 and TLR4 between two and three hours and one and three hours, respectively. After this period of co-culture, there was a decrease in TLR2 and TLR4 expression, concomitant to an increase in the expression of internal receptors such as NLRP3 and NOD2, suggesting phagocytosis of *A. actinomycetemcomitans* and/or its products. 

The engagement of microbial components with TLR2 and TLR4 mediates transcriptional responses through activation of NF-κB, leading to the production of pro-inflammatory cytokines, including TNF-α and inactive pro-IL-1β [39]. Furthermore, the response of macrophages to *A. actinomycetemcomitans* infection was obvious, with immediate and continuous upregulation of *TNF-α*, *IL-1β* and, to a lesser extent, *IL-18*. 

*A. actinomycetemcomitans* phagocytosis by macrophages was impaired due to cytolethal distending toxin production, but the ability to produce nitric oxide (NO) and TNF-α was still functional in the intoxicated macrophages [41]. The upregulation of extra- and intracellular receptors for PAMPS and DAMPS in macrophages reinforced these observations. Binding of PAMPS to TLRs and NOD2 activates the NF-kB pathway, inducing the expression of inflammatory cytokines [42]. When the signaling transduction pathways were analyzed, as shown in Figure 4, the mitogen activated pathway (MAPK) ERK1/2 was activated in *A. actinomycetemcomitans-*infected macrophages, which was suggestive of NF-κB activation after 120 min of incubation, as indicated increased levels of pIkB-α at this time point (Figure 5). The increased level of phosphorylated serine–threonine kinase (pAKT), also observed after 120 min of macrophage interaction with *A. actinomycetemcomitans,* indicated activation of the PI3K/AKT kinase pathway, which indirectly activates NF-κB via proteasome degradation of IκB. The phosphorylation of 4EBP1 at Thr37/46 decreases its association with eIF4E and consequently inhibits the mRNA translation of growth, thereby promoting protein synthesis [43]. Our results demonstrated that in the late co-culture period, there were decreased levels of phosphorylated 4EBP1, suggesting that *A. actinomycetemcomitans* was not able to affect the viability of the macrophages. 

Interestingly, the signaling pathway responses in HGECs were different from those of macrophages, with decreased levels of phosphorylated ERK1/2 and increased levels of 4EBP1 in the phosphorylated form. Additionally, AKT phosphorylation was not observed up to 30 min after interaction of *A. actinomycetemcomitans* with HGECs, which was in accordance with the pro-apoptotic phenotype of epithelial cells reported after 60 min of bacteria–HGEC interaction (Handfield et al., 2005). AKT is key in cellular survival [44] and was only activated when the infected cells were incubated for a prolonged period. Inhibition of the AKT/mTOR pathway in infected macrophages and at early stages of infection in HGECs may exert bacterial clearance effects. AKT inhibition promoted by *Streptococcus pneumoniae* was associated with infection progression and inhibition of autophagy [45,46]. In contrast, attenuation of the AKT/mTor pathway enhanced autophagy and *Salmonella ssp.* clearance [47], indicating that subversion of this pathway may result in different outcomes according to the infecting agent. 

Another studied protein was pc-FOS, which is one of the downstream factors induced by ERK1/2 pathway [48]. c-FOS phosphorylation is involved in osteoclastogenesis, leading to differentiation of precursor cells into osteoclasts due to the production of colony-stimulating factor 1 (CSF-1) and receptor activator of the NF-kB ligand (RANKL) [49]. Although previous data indicated that *A. actinomycetemcomitans* lipopolysaccharide (LPS) induced c-FOS phosphorylation in human gingival fibroblasts [50], our data indicated no increase in pc-FOS after co-culture with live *A. actinomycetemcomitans*, suggesting that ERK1/2 pathway activation did not induce c-FOS phosphorylation in the HGECs or U937 macrophages.

After *A. actinomycetemcomitans* infection, increased levels of IL-1β, TNF-*α* and IL-18 transcripts were observed in macrophages (Figure 1). Activation of NF-κB leads to pro-IL1 production, whereas pro-IL-18 is constitutively expressed but its expression increases after cellular activation [51,52]. The increase in IL-1β levels after one to three hours and in TNF-α levels after prolonged incubation with *A. actinomycetemcomitans-*infected macrophages possibly indicated that the binding of IL-1β to its receptor IL-1R1 led to a cascade of downstream events, eventually resulting in the expression of TNF-α. Silencing of NRLP3 indicated that activation of this intracellular receptor mediates pro-IL-1β and pro-IL-18 production in infected macrophages (Figure 5). However, the release of the mature and bioactive IL-1 family of cytokines, including IL-1β and IL-18 [51,52], is mediated by caspase-1 in inflammasomes [53]. Our data also indicated that *A. actinomycetemcomitans* Y4 infection in macrophages led to cleavage of procaspase-1 to caspase-1(Figure 4 and Figure 5), resulting in the release of active IL-1β. This observation contradicted a previous study which reported that *A. actinomycetemcomitans* Y4 was not able to induce caspase-1 expression in monocytes, despite the increased expression of IL-1β [31]. 

On the other hand, the response of HGECs to *A. actinomycetemcomitans* resulted in a slight increase in transcript levels of *IL-1β* and *IL-18* after one hour of incubation, which returned to levels below those achieved in control cells thereafter (Table 1). Furthermore, the levels of TNF-α and IL-1β in cell supernatants were high in infected macrophages, but not in HGECS, shortly after incubation with *A. actinomycetemcomitans* (Figure 1). These data were in accordance with others reporting that HGEC interaction with *A. actinomycetemcomitans* extracts for eight hours did not result in IL-1β production, and increased levels of this cytokine were observed only after 12 h of incubation [54]. Other data indicated that *A. actinomycetemcomitans* infection leads to upregulation of *IL-1β* in HGECs after 60 min of incubation [55]. However, we showed that this upregulation was not maintained after longer interaction periods and did not result in the production of significant amounts of pro-IL-1β. When we analyzed the TNF levels, we observed a small increase of this cytokine in the presence of *A. actinomycetemcomitans*, however, in the presence of *Porphyromonas gingivalis,* the increase was higher after 24 h of co-culture with HGECs, showing that the cells were responsive to another periodontal pathogen [56]. Despite a limited number of samples of HGCEs from different donors, this study was in accordance with the low inflammatory response in the presence of *A. actinomycetemcomitans* when co-cultured with immortalized gingival cells OBA-9 (unpublished data). Furthermore, differences between this work and other studies regarding the tested *A. actinomycetemcomitans* strain, as well as differences in the origins of the monocytic cells and distinct macrophage phenotypes, may have contributed to conflicting data [31]. Overall, our results were in accordance with those that previously verified that *A. actinomycetemcomitans* and its product, Cdt, were able to induce inflammasome activation in macrophages [30,57]. 

Thus, our data indicated that, although macrophages responded to *A. actinomycetemcomitans* infection by upregulating the expression of intra- and extracellular receptors and production of cytokines (Figure 4), HGEC response to *A. actinomycetemcomitans* was mild, differing from that of other periodontopathogens, such as *P. gingivalis* [36].

Induction of inflammasomes in response to the microbial community was shown to control the microbiota in the gut, whereas its depletion induced dysbiosis [58]. Macrophages promptly recognize *A. actinomycetemcomitans* and its products, activating inflammasomes, which may be important in control of oral dysbiosis, but may also result in tissue destruction and perpetuation of inflammation. However, epithelial cells are the first barrier against pathogens and their response is important for the subsequent immune response. Epithelial cells internalize *A. actinomycetemcomitans* and a pro-apoptotic phenotype is induced by this species [55]. Thus, the low response to the pathogen by epithelial cells may be an additional factor of evasion of host defense mechanisms, facilitating colonization and dissemination to the underlying tissues in oral mucosa. This study demonstrated the differences between the responses of macrophages and gingival epithelial cells in the presence of *A. actinomycetemcomitans.*

## 4. Material and Methods

### 4.1. Eukaryotic Cells and Bacteria Culture Conditions

Gingival tissue was obtained from young healthy adult patients after third molar extractions (HGECs) by approval of the Institutional Review Board [5,56]. HGECs at the 3rd passage were harvested, seeded at a density of 0.5 × 10^5^ cells/well on 6-well plates in KSFM (Keratinocyte Serum Free Medium) medium (Life Technologies, Carlsbad, CA, USA) supplemented with 10 mg/mL insulin, 5 µg/mL transferrin (Sigma-Aldrich, St Louis, MO, USA), 50 µg/mL bovine pituitary extract (BPE) (Life Technologies), 3-factor supplement (10 µM of 2-mercaptoethanol, 10 µM ethanolamine and 10 nM NA-Selenite- Sigma-Aldrich), 1% penicillin–streptomycin solution (Sigma-Aldrich) and 25 µg/L of fungizone (Life Technologies).

The human monocytic cell line U937 [59] was maintained in suspension culture in RPMI-1640 (Life Technologies, Carlsbad, CA, USA) supplemented with 10% (v/v) heat-inactivated fetal bovine serum (FBS) (Hyclone, Logan, UT, USA), 1% penicillin–streptomycin solution (Sigma-Aldrich) and 25 µg/L of fungizone and amphotericin B solution (Gibco, Scotland, UK), at 37 °C in a humidified atmosphere of 5% CO_2_. U937 cells were differentiated into adherent macrophage-like cells by exposure of 4 × 10^6^ cells to 20 nM phorbol 12-myristate 13-acetate (PMA) (Sigma-Aldrich) for 24 h and left to differentiate for an additional 48 h in 5% CO_2_ at 37 °C [60].

*A. actinomycetemcomitans* strain Y4 serotype b was grown under microaerophilic conditions at 37 ℃ in tryptic soy broth (Sigma-Aldrich) supplemented with 0.6% weight/volume yeast extract.

### 4.2. Co-culture Assay

HGECs or differentiated U937 macrophages cultivated in media (5% fetal bovine serum) without antibiotics were infected with *A. actinomycetemcomitans* Y4 cells culture at mid log-phase at an multiplicity of infection (MOI) of 1:100 (eukaryotic cell:bacteria). After incubation for 1, 2, 3, 4 and 8 h for U937 macrophages and HGECs, the supernatants were collected and cells were washed twice with 1x phosphate saline buffer (PBS) prior to total RNA extraction. In order to verify the activation of signaling pathways in *A. actinomycetemcomitans-*infected cells, the co-cultures were obtained as described at time points 0, 15, 30, 60, 90 and 120 min, and the cell lysates were used in a Western blot assay. Noninfected HGECs and U937 macrophages were used as negative controls. The study was performed in three independent experiments and the HGECs were obtained from three different donors.

### 4.3. Gene Expression

Expression of inflammasome-related genes in infected HGECs and U973 macrophages was assessed by reverse transcription followed by real-time PCR (RT-qPCR). Total RNA was extracted from cultured U937 cells and HGECs using the RNeasy mini kit (Qiagen, Hilden, Germany), according to the manufacturer’s instruction. Ten micrograms of RNA was used to obtain first strand cDNA synthesis using the High-Capacity cDNA Archive kit (Applied Biosystems, Foster City, CA, USA) in a total volume of 100 µL. Real-time PCR was performed using an ABI 7500 system (Applied Biosystem). The transcription of genes encoding the receptors NLRP3 (Hs00918082_m1), NOD1 (Hs01036720_m1), NOD2 (Hs01550753_m1), TLR2 (Hs02621280_s1) and TLR4 (Hs00152939_m1) and cytokines IL-1β (Hs01555410_m1), IL-18 (Hs01038788_m1) and TNF-α (Hs00174128_m1) were evaluated using Taqman probes for human and theTaqMan Gene Expression Master Mix (Applied Biosystem). Transcription of human GAPDH (Hs02786624_g1) was used as an endogenous control. Results were analyzed using 2^−ΔΔCt^, where the results were normalized using the housekeeping gene or gene of interest from the reference sample in the case of cells at 0 h and compared with genes of the other samples using the threshold cycle (Ct) values from the real-time reaction [61]. 

### 4.4. Activation of Inflammasome-Related Signaling Pathways

The amount of phosphorylated proteins indicative of activation of different pathways and other proteins involved in inflammasomes were determined in HGECs and U937 macrophages co-cultured with *A. actinomycetemcomitans* by Western blotting. The cells were washed twice with 1x PBS, suspended in 1x SDS-PAGE loading dye (BioRad, Hercules, CA, USA) and boiled. SDS-PAGE was carried out according to the Laemmli method [62]. The gel was transferred to the nitrocellulose membrane (Life Technologies) at 4 °C in a Mini-Trans-Blot cell (Life Technologies) apparatus for 2 h at 370 mA. The primary antibodies were for phosphorylated ERK1/2 (pERK1/2) at T202/Y204, pAKT (Ser 473), p4EBP-1 (Trr 37/46) and pc-Fos (Ser 32). In U937 cells, pIκB-α (Ser32) (Cell Signaling, Danvers, MA, USA), caspase-1 p10 (C20) and cleaved caspase p10 were also evaluated. As a control, the antibody anti-GAPDH (Santa Cruz Biotechnology, Dallas, TX, USA) was used at 1:1000 dilution. The secondary antibody was anti-rabbit IgG, HPR-linked and diluted at 1:2000. Protein detection was performed using Amershan ECL Prime Western Blotting Detection reagent (GE Healthcare, Uppsala, Sweden). After detecting one protein, the primary and secondary antibody detected previously was removed using Restore™ Western Blot Stripping Buffer (Thermo-Scientific) and the same gel was used to detect other proteins. The results are representative of three independent experiments and cells at 0 h were used as the control. 

### 4.5. Cytokines Quantification

Levels of secreted IL-1β and TNF-α were determined in the supernatants of U937 and HGEC that were co-cultured with *A. actinomycetemcomitans* by ELISA using an R&D systems kit (Minneapolis, MN, USA). The plates were read in a microplate reader at an optical density (OD) of 450 nm. The amounts of each cytokine were determined after comparison with the respective standard curve.

### 4.6. Silencing of NLRP3

In order to confirm inflammasome activation by *A. actinomycetemcomitans in* macrophages, silencing of NLRP3 was performed. U937 cells (2 × 10^6^ cells) were transfected with siNLRP3 (Dharmacon-Thermo Scientific, Waltham, MA, USA) using the Amaxanucleofactor kit V (Lonza, Allendale, NJ, USA). Cell suspensions were centrifuged at 90× *g* for 10 min, the medium was removed and 1 µM of siNLRP3 in 100 µL of nucleofactor V buffer were added. As a negative control, scramble siRNA (siControl non-targeting siRNA Pool, Horizon Discovery, UK) was used. The samples were electroporated in nucleofactor program V-001 (Lonza), RPMI medium was added and the cells were transferred into 6-well plates with 2.5 mL of phorbol 12-myristate 13-acetate (PMA) in RPMI medium and incubated for 24 h. Co-cultures of transfected U937 macrophages with *A. actinomycetemcomitans* were performed as described. Transcription of genes encoding NRLP3, IL-1β, IL-18 and TNF-α was determined by RT-qPCR and the production of cytokines in the cell supernatants was measured by ELISA.

### 4.7. Statistical Analysis

Comparisons between samples were performed by one-way ANOVA followed by post-Tukey’s test (Graphpad Prism version 4.0, La Jolla, CA, USA). Results were considered significant when p < 0.05.

## 5. Conclusions

Taken together, our data indicated that *A. actinomycetemcomitans* enhanced the expression of NLRP3, TLR4, TLR2 and NOD2 in macrophages but not in HGECs, consequently inducing distinct signaling pathways and cytokine production and demonstrating varied innate immune responses depending on the cell type.

## Figures and Tables

**Table 1:** Effect of co-culture of *A. actinomycetemcomitans* strain Y4 (Aa- MOI 1:100) with human gingival epithelial cells (HGECs) for 1, 2, 3, 4 and 8 h on the relative transcription of *TLR4, TLR2, NLRP3, NOD1, NOD2, IL-1β, IL-18* and *TNF-α* detected by real-time PCR and expressed in terms of fold-change in comparison with control. Control consisted of infected cells at 0 h. Transcription of the target gene was normalized according to mRNA levels of GAPDH; = data are shown as fold-change ± standard deviation (SD) representative of three independent experiments.

Gene Expression (HGECs)	Control (0 h)	Aa 1 h	Aa 2 h	Aa 3 h	Aa 4 h	Aa 8 h
		Fold-Change (±SD)				
*tlr-4*	1.00	0.79 (±0.14)	0.74 (±0.10)	0.68 (±0.08)	0.39 (±0.04)	0.32 (±0.01)
*tlr-2*	1.00	0.78 (±0.01)	0.66 (±0.09)	0.47 (±0.04)	0.41 (±0.03)	0.46 (±0.04)
*nlrp3*	1.00	1.15 (±0.23)	0.76 (±0.11)	0.64 (±0.09)	0.50 (±0.03)	1.07 (0.05)
*nod1*	1.00	1.15 (±0.24)	1.38 (±0.20)	0.80 (±0.00)	0.58 (±0.02)	0.42 (0 05)
*nod2*	1.00	0.77 (0.09)	0.77 (±0.09)	0.72 (±0.08)	0.77 (±0.01)	0.76 (0.03)
*il-1*β	1.00	1.22 (±0.06) *	0.72 (±0.01)	0.33 (±0.01)	0.51 (±0.02)	0.13 (±0.01)
*Il-18*	1.00	1.27 (±0.12) *	0.93 (±0.09)	0.70 (±0.05)	0.64 (±0.09)	0.43 (±0.02)
*tnf*	1.00	0.88 (±0.05)	0.98 (±0.06)	2.00 (±0.21) *	3.72 (±0.13) *	0.94 (±0.09)

* Statistically significant difference in comparison with control (ANOVA–Tukey’s, p < 0.05).

**Table 2:** Effect of co-culture of *A. actinomycetemcomitans* strain Y4 (Aa- MOI 1:100) with U937 macrophages for 1, 2, 3, 4 and 8 h on the relative transcription of *TLR4, TLR2, NLRP3, NOD1, NOD2 IL-1β, IL-18* and *TNF-α* detected by real-time PCR and expressed in fold-change in comparison with control. Control consisted of infected cells at 0 h. Transcription of target gene was normalized according to mRNA levels of GAPDH; data are shown as fold-change ± SD representative of three independent experiments.

Gene Expression (U937 cells)	Control (0 h)	Aa 1 h	Aa 2 h	Aa 3 h	Aa 4 h	Aa 8 h
		Fold-Change (±SD)				
*tlr-4*	1.00	1.27 (±0.03) *	1.30 (±0.10) *	1.15 (±0.01) *	0.44 (±0.00)	0.25 (±0.00)
*tlr-2*	1.00	1.18 (±0.10)	1.51 (±0.18) *	1.47 (±0.14) *	0.75 (±0.04)	0.68 (±0.01)
*nlrp3*	1.00	0.92 (±0.01)	2.03 (±0.17) *	1.85 (0.18) *	0.70 (±0.02)	0.83 (0.04)
*nod1*	1.00	1.24 (±0.03)	1.07 (±0.05)	1.13 (±0.40)	1.90 (±0.17)	0.76 (0.09)
*nod2*	1.00	1.25 (0.07) *	1.24 (±0.02) *	1.29 (±0.07) *	1.67 (±0.13) *	2.34 (0.06) *
*il-1b*	1.00	10.75 (±0.43) *	31.79 (±0.30) *	43.19 (±1.01) *	26.75 (±2.88) *	54.56 (±7.20) *
*Il-18*	1.00	1.62 (±0.05) *	1.34 (±0.16) *	1.42 (±0.17) *	1.23 (±0.13)	0.83 (±0.01)
*tnf*	1.00	7.18 (±0.41)	85.13 (±6.84) *	134.96 (±26.93) *	85.53 (±1.44) *	68.86 (±2.54) *

* Statistically significant difference in comparison with control (ANOVA–Tukey’s, p < 0.05).
